# Comparative Genomic and Transcriptomic Analysis of Tandemly and Segmentally Duplicated Genes in Rice

**DOI:** 10.1371/journal.pone.0063551

**Published:** 2013-05-16

**Authors:** Shu-Ye Jiang, José M. González, Srinivasan Ramachandran

**Affiliations:** 1 Temasek Life Sciences Laboratory, The National University of Singapore, Singapore, Singapore; 2 Department of Microbiology, University of La Laguna, La Laguna, Tenerife, Spain; University College London, United Kingdom

## Abstract

Tandem and segmental duplications significantly contribute to gene family expansion and genome evolution. Genome-wide identification of tandem and segmental genes has been analyzed before in several plant genomes. However, comparative studies in functional bias, expression divergence and their roles in species domestication are still lacking. We have carried out a genome-wide identification and comparative analysis of tandem and segmental genes in the rice genome. A total of 3,646 and 3,633 pairs of tandem and segmental genes, respectively, were identified in the genome. They made up around 30% of total annotated rice genes (excluding transposon-coding genes). Both tandem and segmental duplicates showed different physical locations and exhibited a biased subset of functions. These two types of duplicated genes were also under different functional constrains as shown by nonsynonymous substitutions per site (*Ka*) and synonymous substitutions per site (*Ks*) analysis. They are also differently regulated depending on the tissues and abiotic and biotic stresses based on transcriptomics data. The expression divergence might be related to promoter differentiation and DNA methylation status after tandem or segmental duplications. Both tandem and segmental duplications differ in their contribution to genetic novelty but evidence suggests that they play their role in species domestication and genome evolution.

## Introduction

Gene duplication is prominent in eukaryotes. More than one-third of protein-coding genes belong to multigene families in model organisms [1], [2]. In rice, based on our preliminary study, around 6,000 gene families were detected to encode more than two thirds of the total annotated non-transposon proteins. Both tandem and segmental duplications significantly contribute to the origin, expansion and evolution of multigene families. Tandemly duplicated genes are located next to the original copy or are separated by several un-related genes. They are presumed to originate through unequal crossing over or transposon activities [3], [4]. Segmentally duplicated genes result from duplications of chromosomal regions ranging from 1 to 400 Kb [5], [6]. They arise from the genomic restructure caused by aberrant inter- or intra-chromosome recombination [7].

The genome-wide identification of tandemly duplicated genes has been carried out in both *Arabidopsis* and rice genomes. In both genomes, tandemly duplicated genes are enriched in genes encoding membrane proteins that function under “abiotic and biotic stress” [8]. In *Arabidopsis*, genome-wide identification of segmentally duplicated genes has been also studied [9], [10]. More than 3,000 pairs of segmentally duplicated genes in rice have been identified [11]. Both tandem and segmental duplicates have significantly contributed to the evolution of large gene families in *Arabidopsis* [10]. The investigation from Hanada et al (2008) gives evidence for the importance of lineage-specific expansion of plant tandem duplicates in the adaptive response to environmental stimuli [12]. Besides *Arabidopsis* and rice, genome-wide identification of tandem or segmental genes was also investigated in a few other genomes [13], [14]. Tandem and/or segmental duplications have significantly contributed to the expansion of some gene families in organisms other than *Arabidopsis* [15]–[19]. Recently, Wang et al (2011) concluded that gene duplication modes contribute differently to genetic novelty and redundancy [20]. However, relatively little has been reported on the comparative analysis of these two duplicate modes in their roles in biological function and species evolution.

What is of particular interest is the fate of duplicated genes. In the classic model a duplicated gene has either lost or gained its new function, which is referred to as pseudogenization or neo-functionalization, respectively [21]. However, based on genomic and transcriptomic data, a much more complex model “duplication– degeneration–complementation (DDC)” was reported [22]. Indeed, the retention mechanisms of duplicated genes were quite diverse [23]–[27]. After gene duplication, one copy might be silenced due to the absence of any selective constraint within the genome [28]. Sometimes gene conversion might play a role in the survival of paralogous genes [29]. Another possibility is that one of the two copies gradually developed a similar new function (sub-functionalization) [30]. A third possibility is that one of the two copies acquires a new function [31]. Tandemly and segmentally duplicated gene pairs provide an excellent genetic collection to study the retention mechanisms of duplicated genes. Both tandem and segmental duplications are originated through completely different mechanisms and the comparison in their functional divergence provides clues to understand their roles in biological evolution and species divergence.

In this study, we first carried out a genome wide identification of all tandemly or segmentally duplicated genes based on the latest version of annotated rice genes. We then examined and compared functional specificities of both tandem and segmental duplicates by gene function enrichment analysis. We also compared and evaluated protein divergence of these two modes of duplicated genes by *Ka/Ks* analysis (where *Ka* = nonsynonymous substitutions per site, and *Ks* = synonymous substitutions per site). In addition, expression divergence among different tissues and genotypes as well as under different abiotic and biotic stresses was investigated and compared to further evaluate their functional divergence after duplication. Finally, we analyzed the effect of promoter similarity and DNA methylation on expression divergence of duplicated genes. Our data showed that the rice genome encodes considerable tandemly or segmentally duplicated genes. Both tandem and segmental duplicates exhibited a biased subset of molecular functions. Both modes of duplicated genes were also under different functional constrains as shown by *Ka/Ks* analysis. Our data imply that these duplicated genes play a role in sub-species diversity in rice.

## Results

### Genome-wide Identification of Tandemly or Segmentally Duplicated Genes in Rice

**Figure 1:**
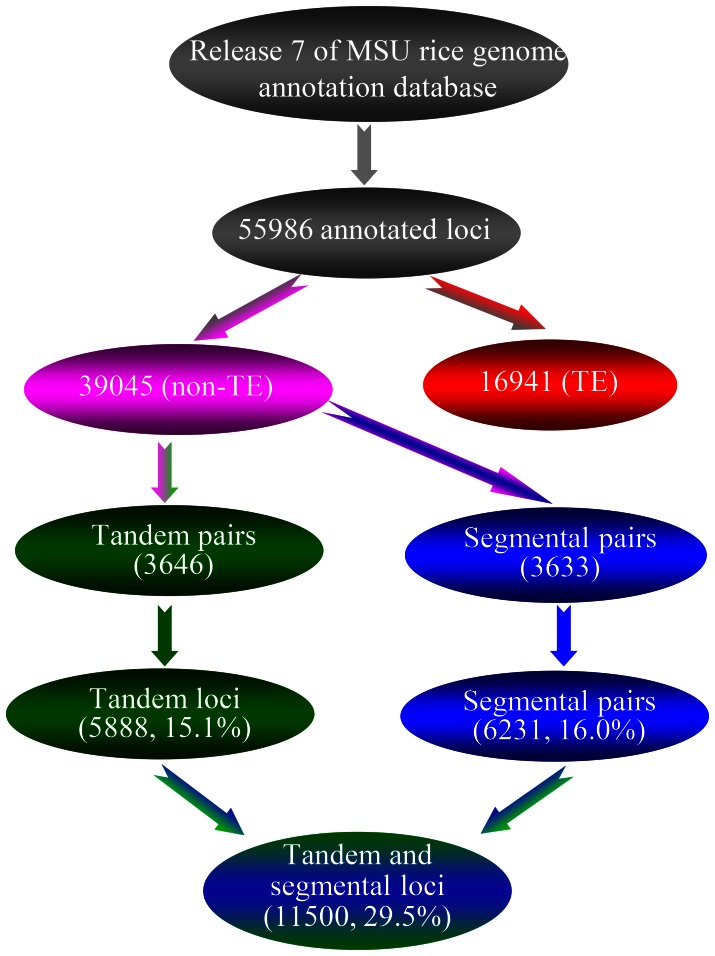
Schematic diagram for identification of tandemly and segmentally duplicated genes. The release 7 of the MSU Rice Genome Annotation Project Database (http://rice.plantbiology.msu.edu/index.shtml) was used for the identification of tandem and segmental genes. Genes encoding TEs were excluded for the identification.

In the latest version (release 7) of the MSU rice genome annotation (http://rice.plantbiology.msu.edu) [32], a total of 55,986 loci were predicted, including 16,941 loci encoding transposon/retrotransposon elements (TEs). Thus, 39,045 genes were predicted to encode non-TE proteins. These genes and their predicted proteins were used for the genome-wide identification of tandemly and segmentally duplicated genes according to the description in the Methods. Based on our searches, we have identified 3,646 pairs of tandem duplicates consisting of 5,888 (15.1%) annotated genes (Figure 1 and Table S1). In addition, a total of 3,633 pairs of segmental duplicates were detected, which consisted of 6,231 (16.0%) annotated genes (Figure 1 and Table S2). Some of the duplicated genes originated from both tandem and segmental duplications. Thus, a total of 11,500 genes were detected, which were involved in either tandem or segmental duplication, accounting for 29.5% of total annotated non-TE genes (Figure 1).

### Distribution of Tandem and Segmental Duplicates on the Chromosomes

**Figure 2:**
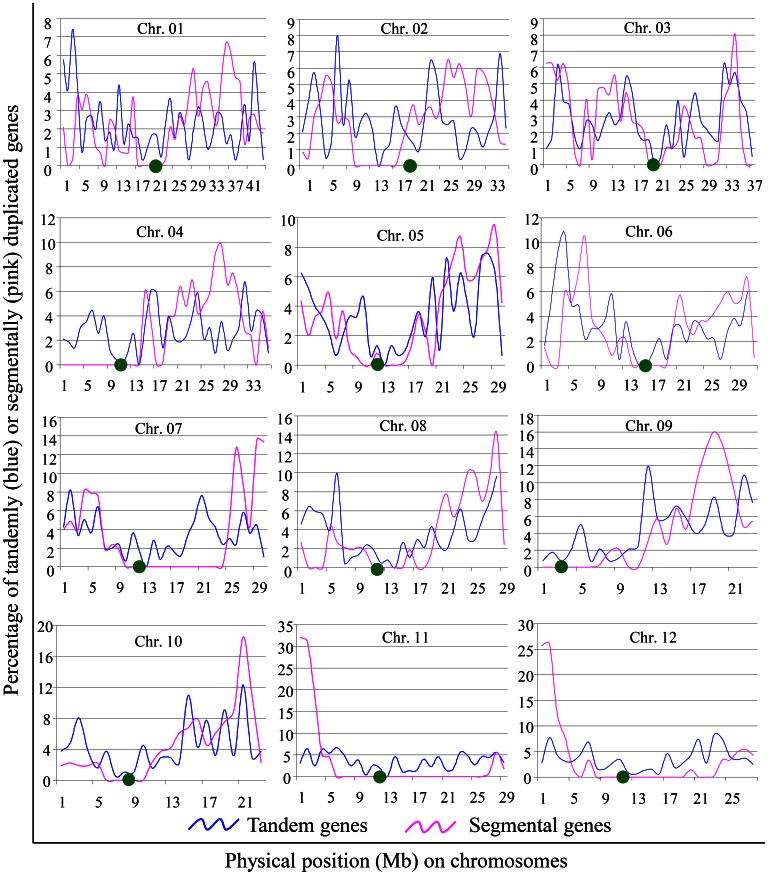
Genomic organization and physical distribution of tandemly and segmentally duplicated genes in the rice genome. Density is indicated by the percentage of the number of tandem/segmental genes in the total number of annotated genes. X-axis indicates chromosomal positions (Mb). Y-axis indicates gene density (the percentage of total number of genes). The distributions of tandem and segmental genes are marked in blue and pink, respectively. Centromere positions are shown with green dots on each chromosome.

On each chromosome, tandem genes ranged from 316 (5.6%) to 838 (14.2%) and segmental genes were from 250 (4.0%) to 844 (13.5%). On chromosomes 1, 4, 6, 8, 9, and 12, similar numbers of tandem duplicates were detected when compared with segmental duplicates (Figure S1). On chromosomes 2, 3 and 5, significantly higher numbers of segmental genes have been detected. However, on chromosomes 7, 10 and 11, a greater ratio of tandem genes were detected. In general, these tandemly and segmentally duplicated genes were not evenly distributed on the 12 rice chromosomes and they exhibited variability in their location on the chromosome (Figure 2). Tandem genes on chromosomes 11 and 12 were distributed evenly except for centromere regions. For other tandem genes, they exhibited uneven distributions with a tendency to cluster near one end or either end of the chromosome (blue curves in Figure 2). Such a tendency was observed more frequently for segmental genes (pink curves in Figure 2). Higher frequencies of segmental genes were observed on the long arm or the end of chromosomes 4, 5, 6, 7, 8, and 10, while the majority of segmental genes were located at the first 5 Mb of chromosomes 11 and 12. Furthermore, overlap distribution of tandem and segmental genes was observed for all chromosomes. However, only 619 genes were detected to have undergone both tandem and segmental duplications. In addition, some chromosomal regions with low frequency of tandem duplication usually showed high frequency of segmental duplication.

### Comparative Analysis of Functional Specificities of Tandemly and Segmentally Duplicated Genes

**Figure 3:**
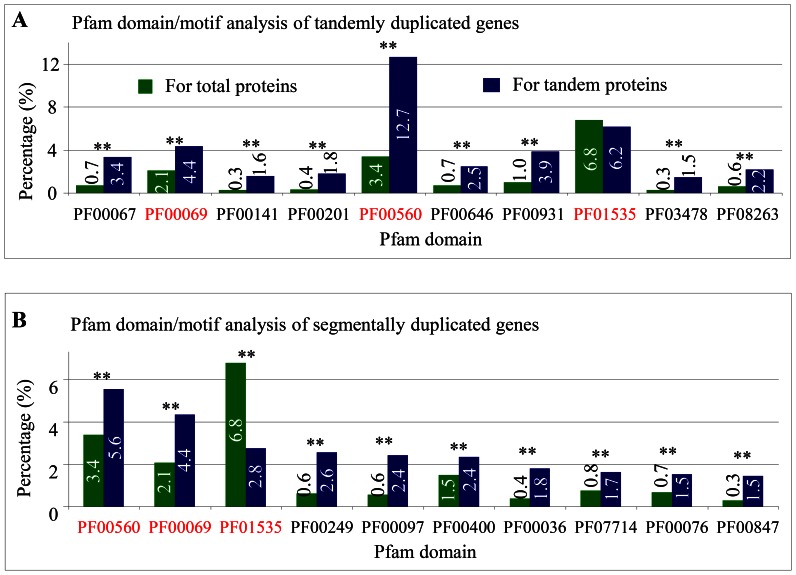
Protein domain analysis in tandemly and segmentally duplicated genes. (**A**) Pfam domain analysis of tandemly duplicated genes. Green and blue columns indicate the percentages of this domain in the total and tandem proteins, respectively. (**B**) Pfam domain analysis of segmentally duplicated genes. Green and blue columns indicate the percentages of this domain in the total and segmental proteins, respectively. Commonly detected domains in both tandem and segmental proteins are highlighted in red. Two stars indicate statistically significant differences at P value <0.01. PF00067, p450 domain; PF00069, Pkinase domain; PF00141, peroxidase family; PF00201, UDPGT family; PF00560, LRR_1 repeat; PF00646, F-box domain; PF00931, NB-ARC domain; PF01535, PPR motif; PF03478, DUF295 family; PF08263, LRRNT_2 family. Percentage was calculated as the frequency of the Pfam domain in tandemly duplicated proteins and the domain percentage in all proteins was used as the control. PF00249, Myb_DNA-binding; PF00097, zf-C3HC4; PF00400, WD40 repeat; PF00036, efhand; PF07714, Pkinase_Tyr; PF00076, RRM_1; PF00847, AP2.

A protein domain/motif, which is usually highly conserved, play important roles in determining protein functions. To investigate functional specificities of tandem and segmental genes, we ran Pfam searches (http://pfam.sanger.ac.uk/) using all duplicated proteins to predict possible domains/motifs. We selected 10 domains/motifs, which were most frequently detected among tandemly or segmentally duplicated proteins for further analysis (Figure 3 A and B). We detected three domains/motifs that were commonly presented in both tandem and segmental proteins. These domain IDs were PF00069 (Protein kinase domain), PF00560 (Leucine Rich Repeat) and PF01535 (Pentatricopeptide repeat). The first two domains were over-represented in both tandem and segmental proteins. The data suggest that these genes encoding protein kinase and leucine-rich repeat might have undergone large expansion mainly by tandem and segmental duplication during evolution. Pentatricopeptide repeat (PPR) is a 35-amino acid sequence motif, which was commonly found in the plant kingdom. Significantly reduced PPR members were duplicated by segmental duplication when compared with tandem duplication (Figure 3 A and B). The data also suggest that the motif containing family members were mainly expanded by other models of duplication or by transposition.

Besides the three domains, both tandem and segmental duplication exhibited differences in the expansion of the other seven domains/motifs. Tandem duplication played a role in the expansion of p450, peroxidase, UDPGT (UDP-glucuronosyltransferase), F-box, NB-ARC (nucleotide-binding adaptor shared by APAF-1, certain *R* gene products and CED-4), DUF295 (Domain of unknown function) and LRRNT_2 (Leucine rich repeat N-terminal) domains/motifs. Many of these domain/motif containing members function under abiotic and biotic stress related biological processes. Segmental duplication significantly contributed to the expansion of Myb_DNA-binding, zf-C3HC4 (Zinc finger, C3HC4 RING-type), WD40 repeat (short ∼40 amino acid motifs, often terminating in a Trp-Asp dipeptide), efhand (a helix-loop-helix structural domain), Pkinase_Tyr (protein tyrosine kinase), RRM_1 (RNA recognition motif) and AP2 (APETALA2/ethylene-responsive element-binding protein) domain/motif containing members. Many of these members encode transcription factors or regulatory proteins. Thus, these over-represented segmental genes might play a role in transcription regulation and signal transduction.

**Figure 4:**
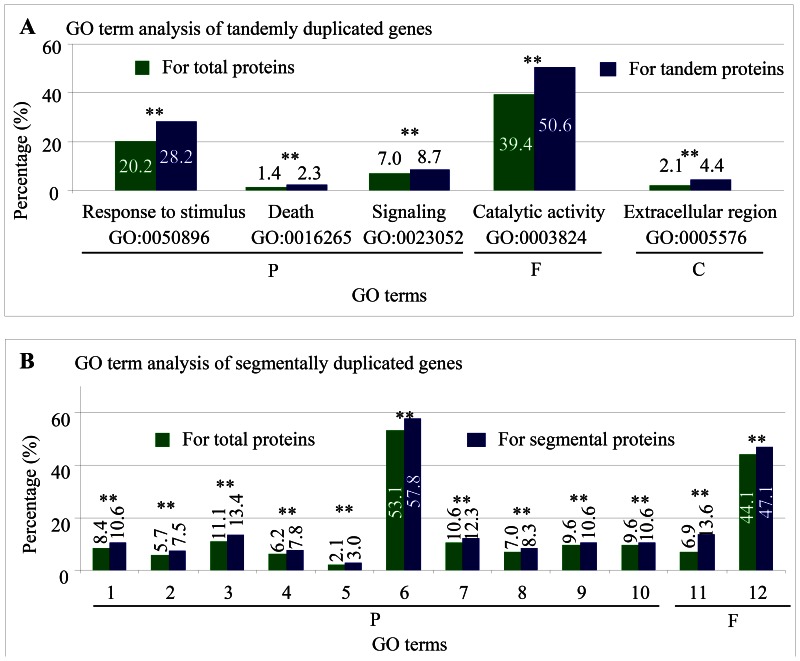
Gene set enrichment analysis of tandemly and segmentally duplicated genes. (**A**) GO term analysis of tandemly duplicated genes. (**B**) GO term analysis of segmentally duplicated proteins. Green and blue columns in (**A**) and (**B**) indicate the percentages of this GO term in total and tandem proteins, respectively. The percentage is calculated as the frequency of the total numbers of each GO term in all proteins with GO term assigned or in all segmentally duplicated proteins with GO term assigned. The two stars in (**A**) and (**B**) represent statistically significant differences at P value <0.01. “P”, “F” and “C” in (**A**) and (**B**) indicate the GO categories biological process, molecular function and cellular component, respectively. GO term annotation in (**B**) refers to the following: 1, GO:0065007 (biological regulation); 2, GO:0023046 (signaling process); 3, GO:0032502 (developmental process); 4, GO:0050789 (regulation of biological process); 5, GO:0040007 (growth); 6, GO:0009987 (cellular process); 7, GO:0032501 (multicellular organismal process); 8, GO:0023052 (signaling); 9, GO:0051234 (establishment of localization); 10, GO:0051179 (localization); 11, GO:0030528 (transcription regulator activity); 12, GO:0005488 (binding).

To further examine the difference in functional specificities between these tandemly or segmentally duplicated genes in rice, we investigated Gene Ontology (GO) terms to identify overrepresented GO terms by GSEA (see Methods). For each term, we identified GO terms in three categories: biological process (P), molecular function (F), and cellular component (C) [33]. Our primary motivation for this analysis was to evaluate whether duplicated genes from different duplication modes were biased toward particular functions. Our data set showed that only one GO term (signaling; GO:0023052; highlighted with red fonts in Figure 4 A and B) was commonly detected in both tandem and segmental genes. Further investigation showed that duplicated tandem genes might play roles in response to stimulus and death (category P), exhibited catalytic activity (category F) and were located in the extracellular region (category C) (Figure 4A). For segmental genes, a total of ten GO terms were detected with over-representation in the category P involved in biological regulation, growth, signaling, localization etc. (Figure 4B). They were also over-represented in transcription regulator and binding activities (category F). The data suggest that different duplication modes produce duplicated genes with biased subsets of biological functions, which might provide the basis for species domestication and genome evolution.

### Protein Divergence after Tandem or Segmental Duplication

**Figure 5:**
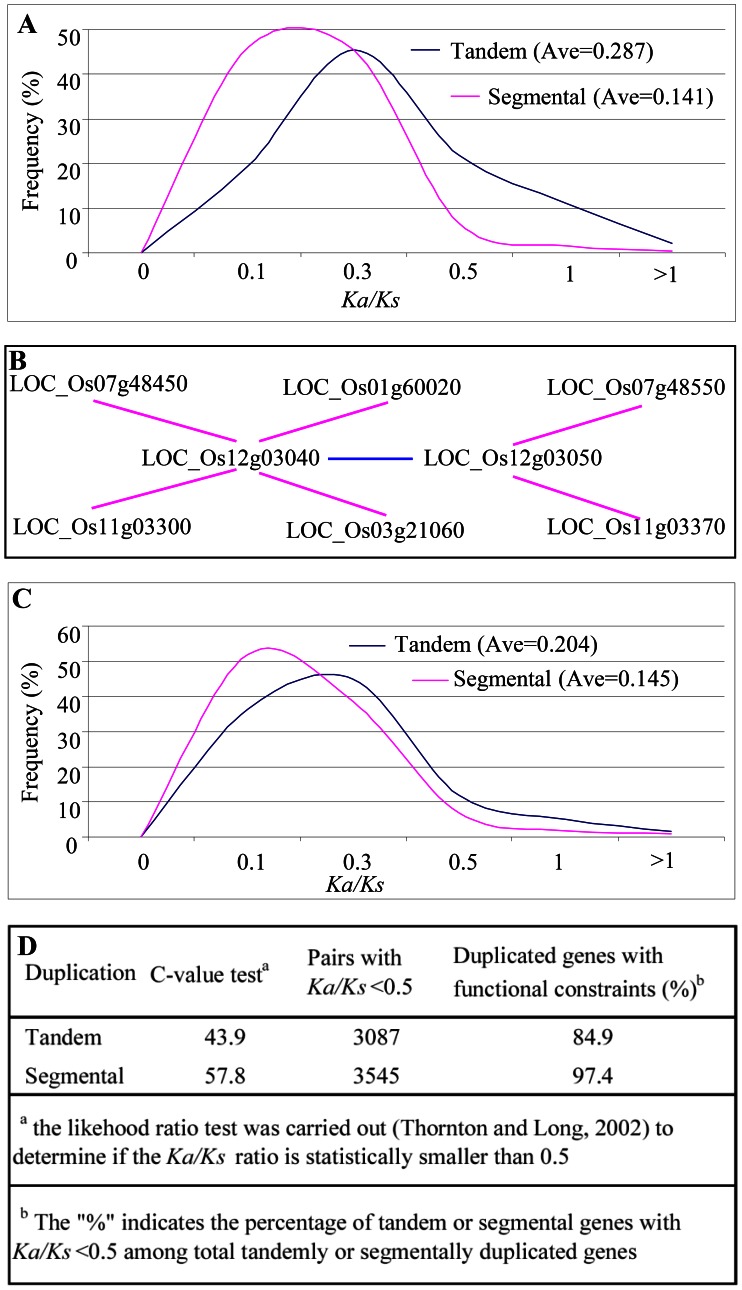
Frequency distributions and C-value test of *Ka/Ks* ratios between gene pairs duplicated from tandem (blue line) or segmental (red line) duplication. (**A**) *Ka/Ks* analysis was carried out using all pairs of tandemly or segmentally duplicated genes. (**B**) An example of genes that have undergone both tandem and segmental duplications. (**C**) *Ka/Ks* analysis using gene pairs from both tandem and segmental duplications. (**D**) Analysis of functional constraints by C-value test.

Tandemly or segmentally duplicated genes accounted for 29.5% of the total annotated non-TE genes. Thus, it would be of interest to know if these duplicated descendants are still functional or have become pseudogenes. The *Ka*/*Ks* ratios of these duplicated pairs were estimated and were tested statistically. Most *Ka/Ks* values for tandemly duplicated pairs were centred near 0.3 (blue line in Figure 5A) with an average *Ka/Ks* ratio of 0.287. For segmentally duplicated genes, most were close to a *Ka/Ks* = 0.2 (pink line in Figure 5A) with an average *Ka/Ks* ratio of 0.141. These data suggest that most of segmental genes have been subject to stronger functional constraints when compared to tandem genes. To further assess the extend of the selective pressure between tandem and segmental duplicates, these genes were submitted to another set of *Ka/Ks* analysis. Such an analysis showed similar results (Figure 5C), further confirming that segmental genes have undergone a higher selective constraint. After tandem/segmental duplication, the *Ka*/*Ks* ratio in a pair could be as low as 0.5 if one gene maintains its original function and the other copy is a pseudogene [34]. Therefore, the ratio of 0.5 was taken as conservative criterion to test the null hypothesis that the ratios are equal to or smaller than 0.5 or greater than 0.5 by C-value test (C = (X-0.5N)/(0.5×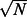), where X is the number of pairs with *Ka/Ks*<0.5 and N is the total number of pairs [34]. We further calculated the C-value to test the null hypothesis that one gene maintains its original function and the other copy is a pseudogene. The calculation showed that C-values of both tandem and segmental pairs are 43.9 and 57.8, respectively (Figure 5D). The results suggested that the probability of the null hypothesis should be very low (P<0.001) for both tandem and segmental pairs (Figure 5D). Up to 84.9% and 97.4% of tandem and segmental genes showed functional constraints (Figure 5D). Thus, most duplicated members are generally under strong selective constraints and both members in each pair should be functional.

### Expression Divergence of Paralogs from Tandem or Segmental Duplication within a Variety

**Figure 6:**
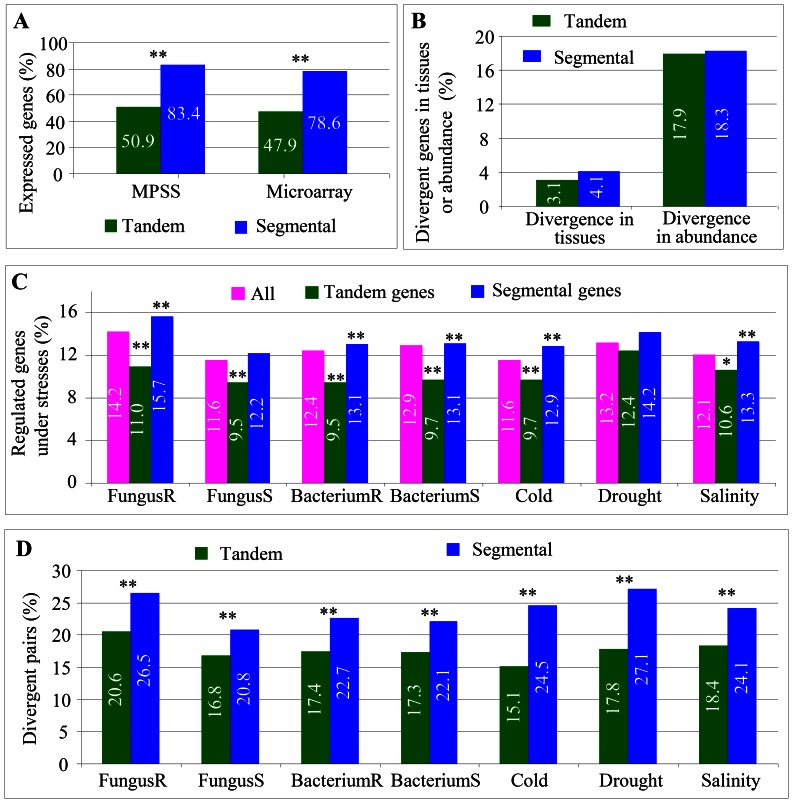
Expression divergence of tandem and segmental genes in Nipponbare. (**A**) Summary of expression profiles of tandem and segmental genes. The analysis was based on MPSS and Affymetrix microarray expression data. Some of the annotated genes were not probed in the array chips. (**B**) Expression divergence of tandem and segmental genes in tissues or abundance. (**C**) Up- or down-regulated tandem and segmental genes under various biotic and abiotic stresses. (**D**) Divergent tandem and segmental pairs in their expression under various biotic and abiotic stresses. In (**C**) and (**D**), FungusR, FungusS, BacteriumR and BacteriumS indicated the biotic stress treatments with incompatible fungus, compatible fungus, incompatible bacterium and compatible bacterium, respectively.

Our data showed that most tandem or segmental genes were under selective constraints. We further analyzed whether these duplicates showed the difference in their expression patterns. We investigated their expression patterns by employing both MPSS and microarray data as described in the Methods. In Nipponbare, only 50.9% of tandem genes were detected with expression signaling by MPSS while up to 83.4% of segmental genes showed expression using the same database (Figure 6A). Similar results were observed when microarray expression data were analyzed in Nipponbare (Figure 6A). Thus, significantly higher percentage of segmental genes was expressed when compared with tandem genes. We then examined expression divergence between tandem or segmental gene pairs in different tissues or their abundance. Around 3.1% or 17.9% of tandem pairs showed difference in their expression patterns among different tissues or their expression abundance (Figure 6B). Similar percentages of pairs were detected with divergence in different tissues or expression abundance for segmental pairs (Figure 6B).

We further investigated their expression regulation of tandem and segmental genes under various biotic and abiotic stresses. For biotic stresses, we analyzed the effects of compatible/incompatible fungi and bacteria on expression. They were labeled as FungusR/FungusS for compatible/incompatible fungus and BacteriumR/BacteriumS for compatible/incompatible bacterium, respectively. On the other hand, we also analyzed expression profiles under various abiotic stresses including cold, drought and high salinity. In general, a smaller percent of tandem genes (green columns) were detected with regulated expression patterns under various biotic and abiotic stresses; however, higher percentages were detected for segmental genes (blue columns) (Figure 6C). For example, under the FungusR stress, we have detected 14.2% of total tested genes with differential expression profiles (pink column in Figure 6C) and the percentage was reduced to only 11.0% for tandem genes (green column in Figure 6C). However, the percentage was increased to 15.7% for segmental genes (blue column in Figure 6C). On the contrary, no difference was detected for the expression regulation under the FungusS stress for segmental genes and under drought stress for both tandem and segmental genes (Figure 6C).

We also examined expression dissimilarities under various biotic and abiotic stresses between tandem or segmental pairs. Interestingly, significantly higher percentages of segmental pairs (blue columns in Figure 6D) exhibited expression divergence under either biotic or abiotic stresses when compared with tandem pairs (green columns in Figure 6D). For segmental pairs, 20.8–27.1% of pairs showed expression divergence while the percentages were reduced to only 15.1–20.6% for tandem pairs (Figure 6D).

### Expression Regulation and Divergence among Genotypes

**Figure 7:**
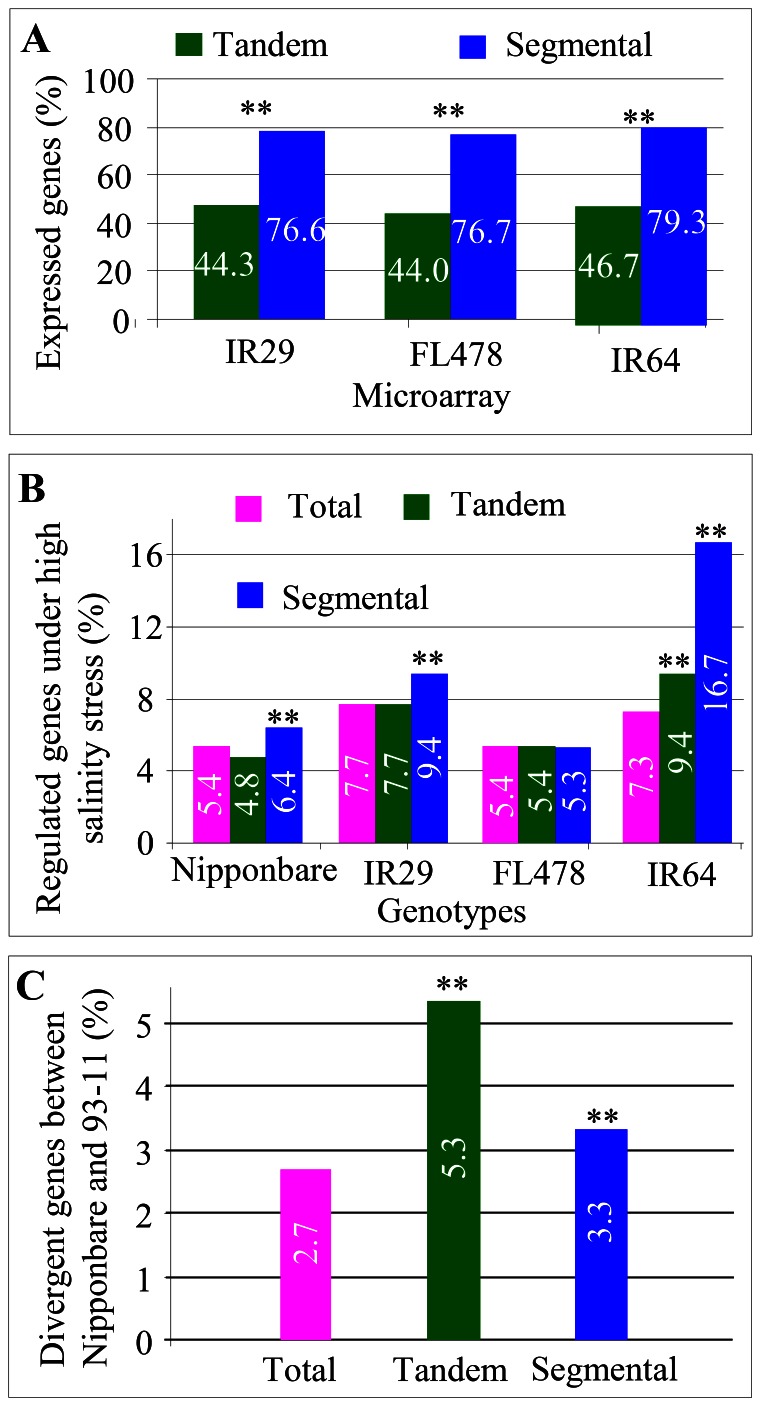
Expression divergence of tandem and segmental genes among different rice genotypes. (**A**) Summary of expressed tandem and segmental genes among three different rice lines including IR29, FL478 and IR64. (**B**) A comparison between tandem and segmental genes in their expression regulation under high salinity stress among four different rice genotypes including Nipponbare, IR29, FL478 and IR64. (**C**) Expression divergence of tandem and segmental genes between Japonica variety Nipponbare and indica variety 93-11.

We have analyzed expression profiles of tandem and segmental genes under normal and stressed conditions in the rice variety Nipponbare. We further examined the expression profiles of these orthologs in other rice genotypes (IR29, FL478 and IR64). Slightly less or similar percentage of expressed genes was detected either for tandem or segmental genes when compared with the data for Nipponbare (Figure 6A and Figure 7A). In addition, a similar trend was observed, that is, significantly higher percentages of segmental genes were expressed when compared with tandem genes. Available data analysis also showed that different genotypes exhibited divergence in the numbers of tandem/segmental genes regulated by high salinity stress (Figure 7B). The highest percentage of segmental genes (16.7%) was observed to be regulated in their expression by the stress. Segmental duplication significantly contributed to expression divergence under high salinity stress in Nipponbare, IR29 and IR64, all of which were salinity-sensitive species. However, in the salinity-tolerance line FL478, no difference was observed (Figure 7B). Genome-wide comparative expression analysis provided a platform to analyze allele-specific expression patterns [35]. We further investigated the expression pattern of tandem or segmental genes in japonica Nipponbare and indica 93-11 (Figure 7C). In total, we detected 2.7% of all genes, which were differentially expressed between these two varieties. However, among tandem or segmental genes, up to 5.3% or 3.3% of the genes, respectively, showed expression divergence (Figure 7C). These data imply a role of tandem and segmental duplication in species divergence.

### Promoter Variation and DNA Methylation in Tandem and Segmental Genes

**Figure 8:**
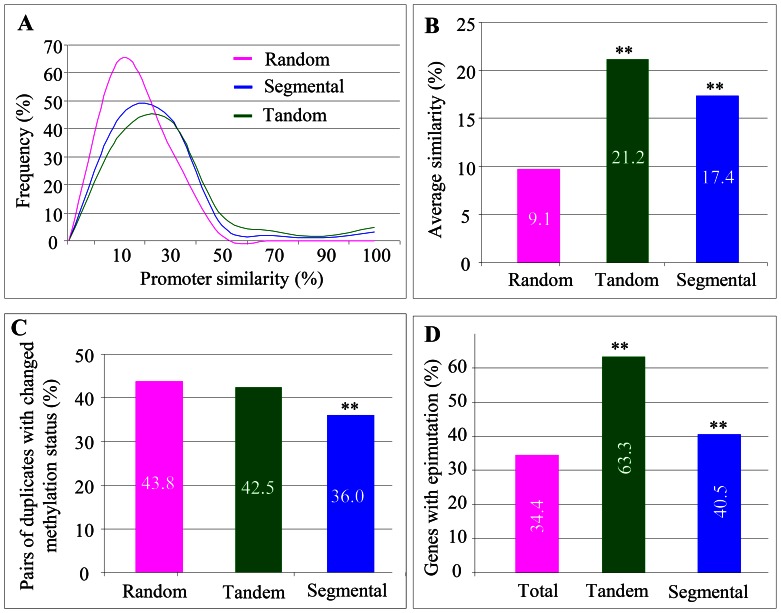
Sequence similarities and DNA methylation status of promoters from tandem and segmental genes. (**A**) Frequency distribution of tandemly and segmentally duplicated promoters by comparing with randomly selected promoters. (**B**) Average similarities of randomly, tandemly and segmentally selected promoters. (**C**) The percentages of promoters with changed DNA methylation status among random, tandem and segmental promoters. (**D**) A comparison in the percentages of genes with epimutation among total annotated, tandem and segmental genes.

Both tandemly and segmentally duplicated genes exhibited significant transcriptional similarities as well as divergences (Figures 6 and 7). The observation prompted us to analyze further their promoter variation after duplication. We determined the promoter similarity by aligning the promoter sequence and comparing these alignments to randomly selected promoter pairs (see Methods). For randomly selected promoter pairs, more than 60% of them had promoter similarity smaller than 10% (pink curve in Figure 8A). For tandem promoter pairs, around 45% of them exhibited the promoter similarity at around 20% (green curve in Figure 8A). For segmental promoter pairs, less than 50% of them had promoter similarity smaller than 20% (blue curve in Figure 8A). In general, the average promoter similarity is only 9.1% for randomly selected promoters, 21.1% for tandem promoters and 17.4% for segmental promoters (Figure 8B). Thus, tandemly duplicated promoters have higher similarity than segmentally duplicated promoters. During tandem and segmental duplication, not only the transcribed regions of genes but also their promoters were duplicated.

Our expression data also showed that some of tandem or segmental gene pairs were detected with expression divergence even though their promoter regions exhibited very high similarity or with 100% homology. To explore further the underlining mechanisms, we investigated the DNA methylation status between tandemly or segmentally duplicated pairs. We determined if a promoter region was methylated according to the description [36]. For randomly selected promoter pairs, up to 43.8% of them showed methylation variation (pink column in Figure 8C). For tandem promoter pairs, the percentage is 42.5% and no significant difference was observed (green column in Figure 8C). However, for segmental promoter pairs, significantly less pairs (36.0%) showed the change in their methylation status (blue column in Figure 8C). In addition, we also analyzed genes that have undergone epimutation according to the method as described [35]. In total, around 34.4% of rice genes were detected to have epimutation (pink column in Figure 8D). However, up to 63.3% and 40.5% of tandemly and segmentally duplicated genes were detected with epimutation, respectively (green and blue columns in Figure 8D). The percentages were significantly higher than the control (34.4%). These data suggest that tandem and segmental genes might have undergone epimutation more frequently.

## Discussion

### Functional Bias of Genes by Tandem and Segmental Duplication and Functional Complementation

In *Arabidopsis* and rice, tandem gene density was positively co-related with the recombination rate [8], [37]. This might be partially due to recombination-mediated processes being involved in tandem duplication [3]. However, our analysis showed that no correlation was observed between segmental gene distribution and recombination rate in rice. Thus, tandem and segmental duplication do not occur at random. Such a duplication mechanism might partially contribute to functional bias of tandem and segmental genes. Tandem arrays were enriched for genes that encoded proteins related to stimulus and death (Figure 4A) but under-represented for genes involved in transcription and DNA/RNA binding [8]. However, segmental duplicates were enriched for genes encoding transcription factors or regulatory proteins (Figure 4B). Thus, our data provided some evidence that shows that tandem and segmental genes might encode genes with functional complementation. The functional bias with a complementation between tandem and segmental genes might be partially determined by the duplication modes. Tandem duplication typically copies one gene each time. Thus, the evolutionarily successful tandem duplication events are most likely to target genes at the end of a pathway, or genes representing flexible steps, such as those involved in environmental response [8]. However, segmental duplication allows for multiple genes to be copied each time, which permits the retention, evolution and divergence of redundant networks.

### Selective Constraints and Mechanisms of Tandem and Segmental Genes

After tandem or segmental duplication, one copy might be silenced or evolve into a pseudogene [28]. Alternatively, both copies might survive under certain selection pressures [38]. Segmentally duplicated genes were generally subject to more stringent functional constraints with an average *Ka/Ks* ratio of 0.141 when compared to tandemly duplicated genes (Figure 5). C-value test further showed that more than 84% and 97% of tandem and segmental genes, respectively, were under functional constraints (Figure 5). The data provided evidence that a limited number of tandemly or segmentally duplicated genes gained novel functions. In this case, it is of interest for us to explain how duplicated genes with similar protein function could have been retained during long evolution. Our data revealed that segmentally duplicated gene pairs showed higher level of expression divergence (Figure 6). This might be due to the promoters from segmental gene pairs having lower level of similarity, thereby, higher promoter divergence when compared with tandem gene pairs (Figure 8). In addition, both tandem and segmental genes also showed the difference in DNA methylation and epimutation (Figure 8). In general, the divergence of protein sequences, transcriptional patterns and abundance as well as DNA methylation status significantly contributed to the retention and evolution of tandemly or segmentally duplicated genes. Higher rate of protein divergence was observed for tandem genes, which might contribute to the retention of tandem genes. In contrast, higher percentage of segmental genes was retained after evolving new transcriptional patterns or abundance with relatively lower protein divergence. In some cases, domain combination was observed in some tandem or segmental genes (data not shown), which might also contribute to the retention of duplicated genes [39], [40]. More expression divergence between tandem or segmental pairs would be expected if expression data from other stress conditions had been available for the analysis.

### Contribution of Tandem and Segmental Duplication to Gene Family Expansion and Species Divergence

Previously, we carried out a genome-wide identification and characterization of several gene families including Lectin [16], GST [18], GRAM [40], and WRKY [41]. The family size ranges from 17 to 267 in the rice genome and both tandem and segmental duplication significantly contributed to their expansion (Table S3). Besides the families mentioned above, both duplications also contributed to the expansion of other families. For example, a total of 687 genes were identified to encode the F-box domain [42]. Based on our analysis, 247 (36%) and 62 (9%) of them were related to tandem and segmental duplication, respectively. These data suggest that both tandem and segmental duplication contribute to the gene family expansion.

During long evolution, some families exhibited lineage-specific expansion through tandem or segmental duplication. Such an expansion formed the basis for adaptive evolution and provided important sources for organizational and regulatory diversity in plants. Shiu et al. (2004) reported a two fold larger RLK/Pelle family in rice than in *Arabidopsis* and tandem duplication seems to be the major mechanism for recent expansions in rice [15]. Their data showed that most of the recent expansions have involved defense/resistance-related genes [15]. Hanada et al. (2008) reported the importance of lineage-specific expansion of plant tandem duplication in the adaptive response to environmental stimuli [12]. Our domain analysis and GSEA data suggest a functional bias of tandem and segmental genes after duplication (Figures 3 and 4). Thus, lineage-specific expansion of some gene families followed by functional bias might significantly contribute to genome evolution and diversity.

The *Ka/Ks* analysis of both tandem and segmental pairs showed obvious functional constraints in their protein sequences (Figure 5). However, functional divergence of small part of tandem or segmental pairs were also observed with *Ka/Ks*>1 (Figure 5). In addition, we also observed considerable tandem and segmental genes with expression divergence within and between genotypes (Figures 6 and 7). Other studies provided evidence that tandem or segmental genes practiced substantial divergence in the expression abundance or tissue specificity or in the response to various abiotic and biotic stresses [12], [18], [19], [41], [43], [44]. On the other hand, tandem or segmental genes also exhibited the divergence in promoter sequences and methylation status (Figure 8). All these data demonstrate the contribution of tandem and segmental genes in variety domestication and species diversity.

## Materials and Methods

### DNA and Protein Data

The release 7 of rice pseudomolecules and protein data were downloaded from the MSU Rice Genome Annotation Project Database (http://rice.plantbiology.msu.edu/index.shtml) [32]. These pseudomolecules are identical to those from the International Rice Genome Sequencing Project (IRGSP, http://rgp.dna.affrc.go.jp/IRGSP/) or the Rice Annotation Project (RAP, http://rapdb.dna.affrc.go.jp). In the release 7, a total of 66,433 gene models were predicted to encode 17,314 TEs and 49,119 non-TEs. A gene may have multiple gene models due to alternative splicing. These gene models were from 55,986 genes including 16,941 loci for TEs and 39,045 loci for non-TEs.

### Identification of Tandemly and Segmentally Duplicated Genes and their Distribution on the Chromosomes

A total of 49,119 non-TE peptides were used for identification of tandemly duplicated genes. Only the longest peptide was retained if multiple peptides were annotated from a same gene locus. Protein sequences were screened in an all versus all BLAST searches using BLOSUM62 matrix and an E-value <0.01. A pair of matching peptides were retained when the identity was > = 30% and the alignment covered > = 70% of the protein length. Pairs of matching proteins were clustered into groups (families) using a transitive closure algorithm: if A = B and B = C, then A = C. Two genes were regarded as tandem pairs if they belonged to the same family, were located on the same chromosome and were separated by no more than 10 unrelated genes.

Segmentally duplicated genes were identified according to the description by Lin et al. (2006) using the release 7 of rice genome dataset (http://rice.plantbiology.msu.edu/index.shtml) [11].

To study the density of tandemly and segmentally duplicated genes, chromosome sequences were split into 1 Mb partitions. Density was calculated for each partition by calculating the percentage of number of tandem/segmental genes among total annotated genes.

### Domain Analysis

All annotated proteins were submitted to the Pfam family database (http://pfam.sanger.ac.uk/) [45] for domain detection. We studied all domains detected in tandem or segmental proteins. For each domain, we calculated the percentage of the domains represented in the tandem and segmental proteins or among the total proteins. We determined whether these two proportions were equivalent by Pearson’s *χ^2^* test.

### GO Annotation and Gene Set Enrichment Analysis

GO assignments for rice genes were obtained from the MSU dataset (http://rice.plantbiology.msu.edu/index.shtml). Three top GO categories (B, F and C) [46] were analyzed. Gene Set Enrichment Analysis (GSEA) [47] was used to determine if a GO category was over-represented in tandem or segmental genes by comparing the partition of the GO category in all annotated rice genes with nominal p-value <0.05 and false discovery rate (FDR) <0.25.

### *Ka/Ks* Analysis and C-value Test

Firstly, amino acid sequences from tandem or segmental pairs were aligned using the “water” program (Smith-Waterman local alignment of sequences, http://emboss.bioinformatics.nl/). The aligned sequences were then transferred to the original coding sequences using the PAL2NAL program [48]. The aligned coding sequences were used for *Ka* and *Ks* estimation by the yn00 program of the PAML4.6 package (http://abacus.gene.ucl.ac.uk/software/paml.html) [49]. The *Ka*/*Ks* ratios were then used to evaluate the protein divergence by testing the C-value according to the description [34].

### Expression and DNA Methylation Analysis

Both massively parallel signature sequencing (MPSS) [50] and Affymetrix rice microarray data were used for expression analysis of tandem and segmental genes. The MPSS expression data were downloaded from the website http://mpss.udel.edu/rice/mpss_index.php. The Affymetrix microarray data were downloaded from the GEO dataset (http://www.ncbi.nlm.nih.gov/geo/) with accession numbers GSE13735, GSE14300, GSE14403, GSE17002, GSE27064, GSE28124, GSE3053, GSE4438, GSE6893, GSE6901, GSE7951. The experiments covered all available Affymerix microarray data under different tissues or under cold, drought and high salinity stresses. A total of 11 tissues were included for the expression divergence: crown vegetative meristematic tissue, germinating seed, germinating seedlings, immature panicle, mature leaves, mature pollens, mature roots, merismatic tissue, stem, young leaves and roots. A duplicated tandem or segmental pairs was regarded as expression divergence in tissues if they showed the difference in detectable tissue numbers. For the transcriptionally detectable tissues in duplicated pairs, if they showed at least two folds difference in their expression abundance in at least one tissue with statistic analysis, they were also regarded as divergent gene pairs. A similar procedure was also applied to the detection of duplicated pairs with down- or up-regulated duplicates. A total of two biotic and three abiotic stresses were analyzed. For biotic stresses, expression data from compatible (S)/incompatible (R) bacteria and fungi were employed including *X.oryzae*-S, *X.oryzae*-R, *M. grisea*-S and *M. grisea*-R. For abiotic stress, we analyzed the expression data under the treatment with cold, drought and high salinity stresses.

For DNA methylation analysis, data sets were downloaded from the GEO dataset (http://www.ncbi.nlm.nih.gov/geo/) with accession numbers GSE21152 and GSE38480 and were analyzed according to their description [35], [51].

### Promoter Similarity Analysis

A total of 3,647 and 3,634 pairs of tandemly and segmentally duplicated promoter sequences (1 Kb upstream of starting code ATG) were aligned using the “matcher” program (Waterman-Eggert local alignment of two sequences, http://emboss.bioinformatics.nl/). Similar pairs of randomly selected rice promoter sequences were also aligned using the same program as a control. For each pairwise alignment, the promoter similarity was calculated as the length of the alignment divided by their total length. Statistic analysis was carried out to evaluate the promoter similarities according to the description [52].

## Supporting Information

Figure S1 **Tandemly or segmentally duplicated genes in each rice chromosome.**
(PPT)

Table S1 **Genome-wide identification of tandemly duplicated genes in the rice genome.**
(XLS)

Table S2 **Genome-wide identification of segmentally duplicated genes in the rice genome.**
(XLS)

Table S3 **Contribution of tandem and segmental duplication to the gene family expansion.**
(XLS)
