# Supplementary material for: Comparative Genomic and Transcriptomic Analysis of Tandemly and Segmentally Duplicated Genes in Rice
Source: PLoS One. 2013 May 16;8(5):e63551. doi: 10.1371/journal.pone.0063551 (PMC3656045; doi:10.1371/journal.pone.0063551)
Supplement: Figure S1 — Tandemly or segmentally duplicated genes in each rice chromosome. (PPT) [file pone.0063551.s001.ppt]

## Slide 1
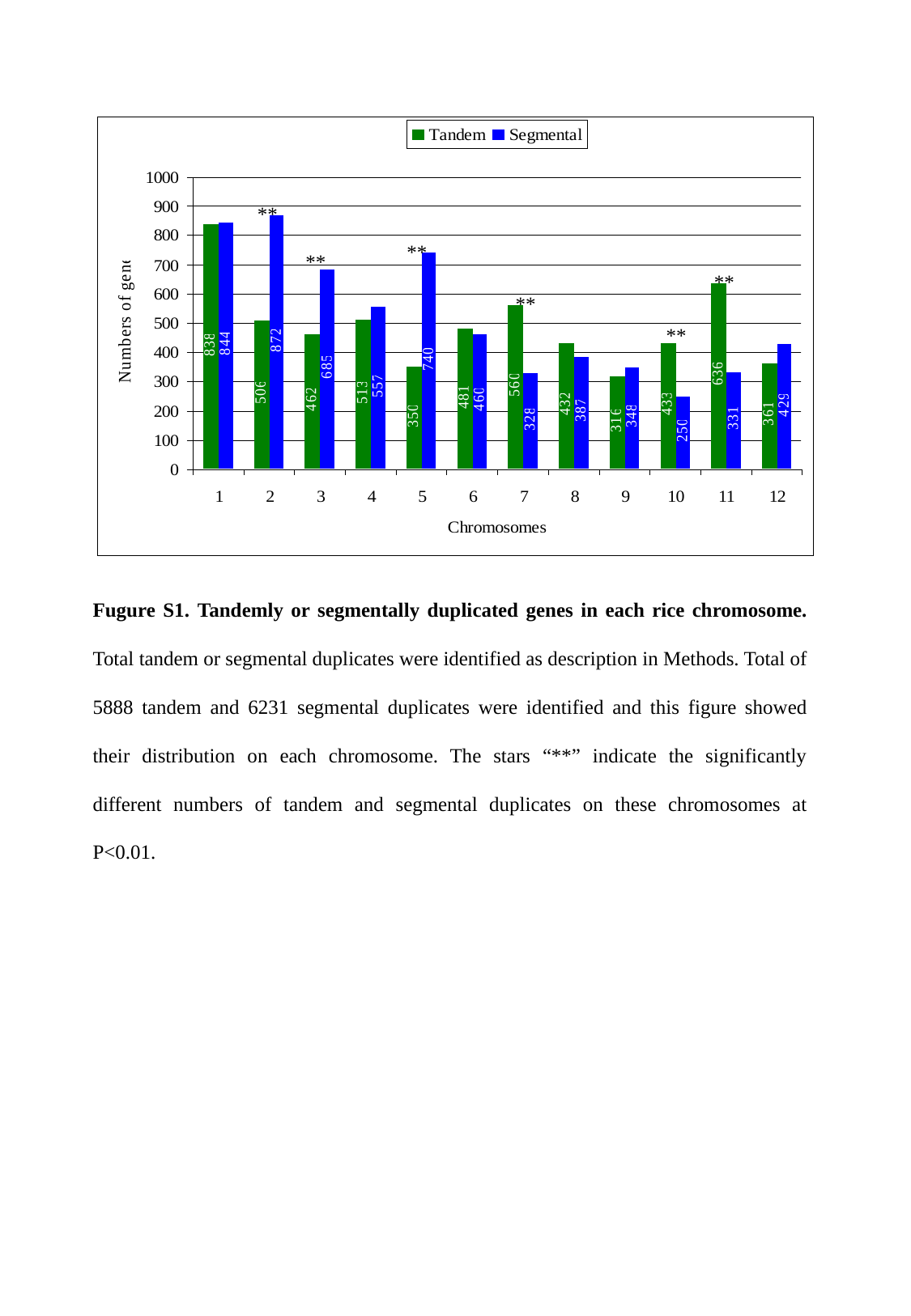

**
**
**
**
**
**
Fugure S1. Tandemly or segmentally duplicated genes in each rice chromosome. Total tandem or segmental duplicates were identified as description in Methods. Total of 5888 tandem and 6231 segmental duplicates were identified and this figure showed their distribution on each chromosome. The stars “**” indicate the significantly different numbers of tandem and segmental duplicates on these chromosomes at P<0.01.
